# Syndecan Family Gene and Protein Expression and Their Prognostic Values for Prostate Cancer

**DOI:** 10.3390/ijms22168669

**Published:** 2021-08-12

**Authors:** Nilton José Santos, Caroline Nascimento Barquilha, Isabela Correa Barbosa, Rodrigo Tavares Macedo, Flávio Oliveira Lima, Luis Antônio Justulin, Guilherme Oliveira Barbosa, Hernandes F. Carvalho, Sérgio Luis Felisbino

**Affiliations:** 1 Department of Structural and Functional BIology, Institute of Bioscience of Botucatu (IBB), São Paulo State University, Botucatu 18618-689, SP, Brazil; nilton.unesp@gmail.com (N.J.S.); caroline.barquilha@gmail.com (C.N.B.); isabela.c.barbosa@unesp.br (I.C.B.); l.justulin@unesp.br (L.A.J.); 2 Department of Structural and Functional Biology, Institute of Biology (IB), UNICAMP—State University of Campinas, Campinas 13083-970, SP, Brazil; gbarbosa.bio@gmail.com (G.O.B.); hern@unicamp.br (H.F.C.); 3 Botucatu School of Medicine (FMB), São Paulo State University, Botucatu 01049-010, SP, Brazil; rodrigotmacedo@gmail.com (R.T.M.); flavio.lima@unesp.br (F.O.L.)

**Keywords:** syndecan, prostate cancer, outcome, prognostic marker, survival, gene expression

## Abstract

Prostate cancer (PCa) is the leading cause of cancer-associated mortality in men, and new biomarkers are still needed. The expression pattern and protein tissue localization of proteoglycans of the syndecan family (SDC 1–4) and syntenin-1 (SDCBP) were determined in normal and prostatic tumor tissue from two genetically engineered mouse models and human prostate tumors. Studies were validated using SDC 1–4 and SDCBP mRNA levels and patient survival data from The Cancer Genome Atlas and CamCAP databases. RNAseq showed increased expression of *Sdc1* in *Pb-Cre4/Pten^f/f^* mouse Pca and upregulation of *Sdc3* expression and downregulation of *Sdc2* and *Sdc4* when compared to the normal prostatic tissue in *Pb-Cre4/Trp53^f/f^-;Rb1^f/f^* mouse tumors. These changes were confirmed by immunohistochemistry. In human PCa, SDC 1–4 and SDCBP immunostaining showed variable localization. Furthermore, Kaplan–Meier analysis showed that patients expressing SDC3 had shorter prostate-specific survival than those without SDC3 expression (log-rank test, *p* = 0.0047). Analysis of the MSKCC-derived expression showed that *SDC1* and *SDC3* overexpression is predictive of decreased biochemical recurrence-free survival (*p* = 0.0099 and *p* = 0.045, respectively), and *SDC4* overexpression is predictive of increased biochemical recurrence-free survival (*p* = 0.035). SDC4 overexpression was associated with a better prognosis, while SDC1 and SDC3 were associated with more aggressive tumors and a worse prognosis.

## 1. Introduction

Prostate cancer (PCa) is the leading cause of cancer-associated mortality in men worldwide [1]. The incidence of PCa in the global population may reflect increased life expectancy, improvements in the health information system, and screening practices using the prostate-specific antigen (PSA) test [2]. Despite advances in the detection of PCa, the main challenge is the difficulty in the early distinguishing of aggressive tumors from indolent ones in patients with a low-grade Gleason score [3]. Promising diagnostic and patient risk stratification approaches to prostate tumor cells have emerged, such as liquid biopsies and exosomes [4,5]. However, new biomarkers are still needed to improve both earlier diagnosis of PCa and patient stratification risk.

The syndecan (SDC) family consists of four transmembrane type I proteoglycans, syndecan-1, -2, -3, and -4 (SDC1–4), which are encoded by four different genes [6,7]. SDCs are differentially expressed in various tissues. SDC1 is found predominantly on the basolateral surface of epithelial cells [8,9]. SDC2 is mainly present in cells of mesenchymal origin, fibroblasts, and endothelial cells. SDC3 is expressed primarily by neuronal tissue and cartilage [10], and SDC4 is found in most tissues but has a relatively low abundance [11,12,13]. SDCs have been associated with cellular signaling, cell adhesion, migration, and exosome release [6,14,15,16].

Syndecans have a six-domain organization. The extracellular amino terminal is modified with glycosaminoglycan chains. All four family members have heparan sulfate changes. SDC1 and SDC3 have additional chondroitin sulfate chains. Proximal to the plasma membrane is a protease-sensitive cleavage site responsible for the shedding of the amino terminal glycosaminoglycan-bearing domain [16,17]. Following the transmembrane domain are three cytosolic carboxy-terminal domains. The highly conserved C1 and C2 domains are intercalated by a variable region. Multiple interactions with both the extracellular matrix and soluble cytokines and chemokines are mediated by the extracellular domain. The transmembrane domain is involved in homodimerization. The cytosolic conserved C1 domain is involved in interacting with the actin filaments via binding to ezrin, radixin, and moesin proteins. In its turn, the C2 domain interacts with synectin, syntenin, and calcium-calmodulin-associated serine/threonine kinase [16,18,19]. Readers are referred to references [19,20] for detailed reviews on syndecans.

The syndecans play an important role in the progression and prognosis of many types of cancer [21,22,23]. SDC1 is the best-characterized member of the SDC family and is expressed in all types of epithelial cells [18,24,25]. Loss of SDC1 is associated with tumor progression and poor prognosis in a variety of cancers [26,27,28]. However, observations described by Palaiologou et al. (2014) showed contradictory results in prostate, breast, ovarian, liver, and pancreatic cancer because SDC1 immunoexpression may change depending on tumor stage [29]. Additionally, syntenin-1 (syndecan binding protein, SDCBP or MDA9), which is important for syndecan signaling, has also been involved in cancer progression. SDCBP shows increasing expression in tumor progression from localized to metastatic lesions [30,31]. As mentioned above, SDCBP binds to the conserved syndecan C2 via its PDZ domains [31]. There is no information on the affinity of SDCBP to the different syndecans.

A consensus on the role of SDCs in human PCa is lacking [25,32,33,34,35,36]. In addition, no study has described the expression pattern of the four SDCs and SDCBP in normal and tumor prostate tissue in both experimental animals and human prostate samples. Therefore, this study aimed to ascertain the gene expression pattern of proteoglycans of the SDCs family and SDCBP in normal and tumoral prostatic tissue of mice and humans.

## 2. Results

### 2.1. Sdc Family Members and Sdcbp mRNA Levels in GEMM of PCa

**Figure 1:**
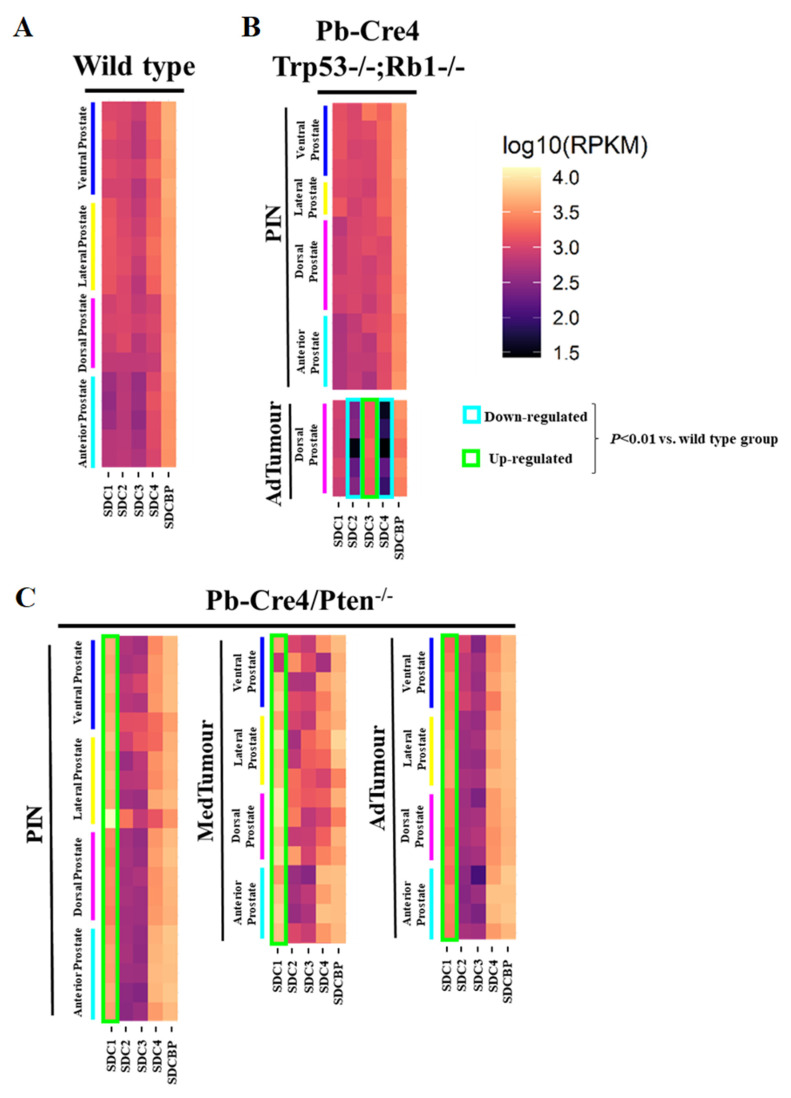
Heatmaps illustrate RNA-Seq differential expression data of the *Sdc1*, *Sdc2*, *Sdc3*, *Sdc4,* and *Sdcbp* genes across different prostatic lobes, mouse models, and tumor stages. (**A**) Non-neoplastic tissue (*Pb-Cre4*-negative—wild type) controls; (**B**) *Pb-Cre4/Trp53^f/f^;Rb1^f/f^* double conditional knockout mouse (p53/Rb mouse); (**C**) *Pb-Cre4/Pten^f/f^* mouse (Pten mouse). The heatmaps represent Log_10_ of normalized RPKM values. PIN Stage—prostatic intraepithelial neoplasia; MedTumor—medium-stage tumor, micro-invasive adenocarcinomas; AdTumor—tumor in a more advanced stage, invasive adenocarcinomas. Note that significant upregulation of *Sdc1* in the Pten mouse (*** *p* < 0.0001 vs. control group), a significant upregulation of *Sdc3,* and downregulation of both *Sdc2* and *Sdc4* in advanced tumors from the p53/Rb mouse were also observed (*p* < 0.0001 vs. wildtype group).

Reads per kilobase of transcript per million mapped reads (RPKM) for each lobe were grouped in a heatmap by condition and stage of progression to facilitate data visualization. Gene expression analysis from RNA sequencing data of two GEMM of PCa showed several essential changes in *Sdc1–4* in prostate tumors at different stages of tumor progression. The level of *Sdcbp* expression did not differ significantly between stages of progression (Figure 1).

In non-tumor tissues, *Sdc1*, *Sdc2*, *Sdc3*, *Sdc4*, and *Sdcbp* were expressed in all four prostate lobes, with no significant quantitative difference among them. Only slightly lower expression of all five genes was observed in the AP than in the other three lobes (DP, LP, and VP) (Figure 1A).

No significant alteration was observed in the p53/Rb mouse. However, a substantial reduction in expression of *Sdc2* was shown in advanced tumor stages in the p53/Rb mouse. No alteration was observed in the expression of the *Sdc3* gene in the Pten mouse, although in the p53/Rb mouse, *Sdc3* overexpression was observed. In the p53/Rb mouse, in advanced tumor stages, *Sdc4* expression was significantly reduced. No significant alteration was observed in the *Sdcbp* gene expression pattern (Figure 1B).

In the Pten mouse, the number of RPKM for *Sdc1* was compatible with increased expression across stages of tumor progression, in PIN lesions, and at medium and advanced stages (Figure 1C).

### 2.2. SDC Family and SDCBP Protein Expression in Knockout Mice Prostatic Tissues

**Figure 2:**
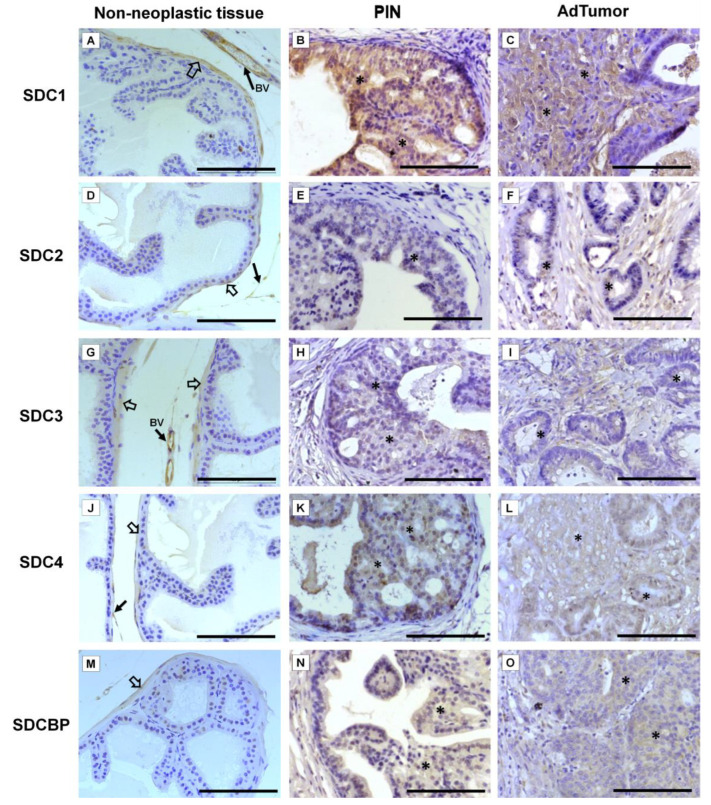
Representative images of the immunohistochemical staining for SDC1, -2, -3, and -4, and SDCBP in non-neoplastic tissues (*Pb-Cre4*-negative—wild type) and tumoral prostatic lobes from *Pb-Cre4/Pten^f/f^* genetic engineered mouse model. (**A**–**C**) SDC1. (**D**–**F**) SDC2. (**G**–**I**) SDC3. (**J**–**L**) SDC4. (**M**–**O**) SDCBP. Prostatic intraepithelial neoplasia (PIN). Advanced tumors (AdTumor). In the non-neoplastic prostatic lobes, positive immunostaining was observed in the interstitial connective tissue (solid arrows), blood vessels (BV), and smooth muscle cells (open arrows). A weak or negative reaction was observed in the secretory epithelial cells for all SDCs and SDCBP. At the PIN and advanced stages of the tumor (asterisks), strong immunostaining for SDC1 in the tumoral epithelial cells, weak immunostaining for SDC4 and SDCBP, and negative immunostaining for SDC2 and SDC3 were observed. Scale bars: 100 μm.

**Figure 3:**
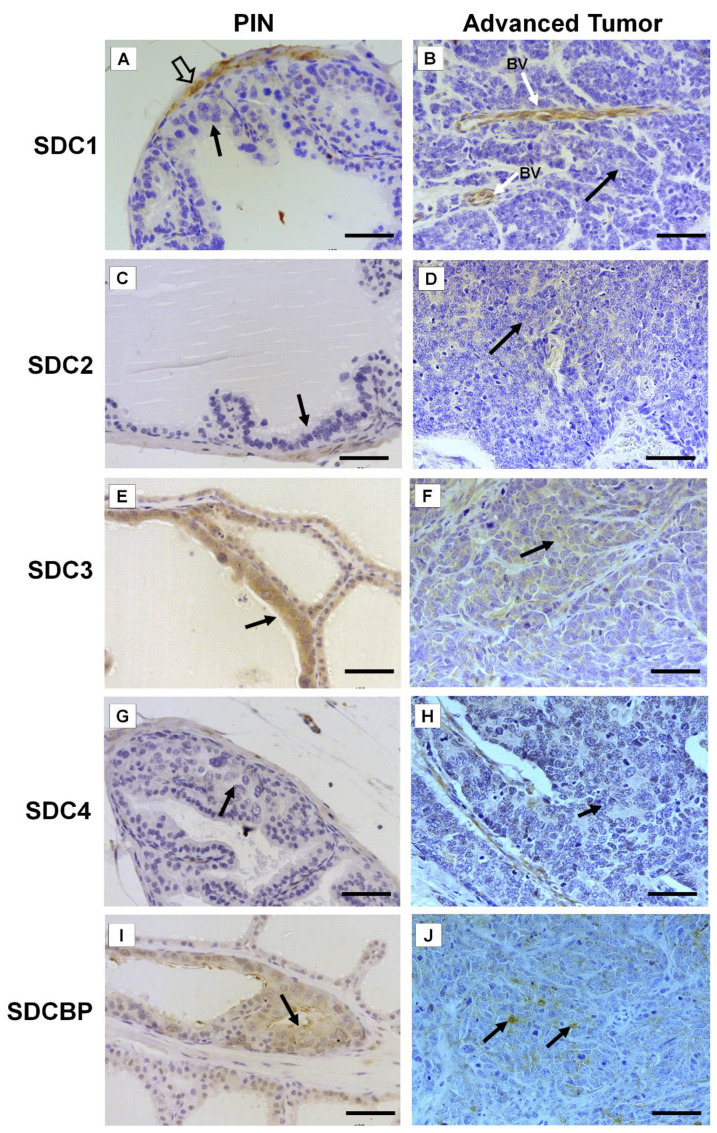
Representative images of the immunohistochemical staining for SDC1, -2, -3, and -4, and SDCBP in tumors found in the different prostatic lobes from the Pb-Cre4/Trp53f/f-;Rb1f/f genetically engineered mouse model. (**A**,**B**) SDC1; (**C**,**D**) SDC2; (**E**,**F**) SDC3; (**G**,**H**) SDC4; (**I**,**J**) SDCBP. Prostatic intraepithelial neoplasia (PIN) and advanced tumors (AdTumor). In the PIN areas and advanced tumors, positive immunostaining was observed for SDC3 (**E**) and SDCBP (**I**) (solid arrows). Blood vessels (BV); smooth muscle cells (open arrow). Scale bars: 100 μm.

Immunohistochemistry for SDC1-4 and its cytoplasmic anchoring protein, SDCBP, were performed on samples from different tumors. The non-tumoral prostatic tissue showed slight epithelial staining and intense staining around smooth muscle cells (Figure 2A). In the Pten mouse, strong immunostaining of SDC1 in the glandular epithelium in the PIN stage (Figure 2B) and in advanced undifferentiated tumors (Figure 2C) was observed. In the p53/Rb mouse, SDC1 was not observed in prostate stromal immunostaining in the PIN stage (Figure 3A) or advanced tumor stage (Figure 3B).

SDC2 showed slight immunostaining in both Pten and p53/Rb mice for both the glandular epithelium and stroma in the PIN and advanced tumor stages (Figure 2D–F; Figure 2C,D). In the normal prostate (non-neoplastic tissue), SDC3 showed strong stromal immunostaining with a marked concentration around blood vessels (Figure 2G). SDC3 showed no reaction in the glandular epithelium of the PIN stage in the Pten mouse (Figure 2H), but SDC3 was poorly expressed in well-differentiated advanced tumors (Figure 2I). In the p53/Rb mouse, SDC3 immunostaining was predominant and intense in the glandular epithelium of PIN lesions (Figure 3E) and moderate in neoplastic cells of advanced tumors (Figure 3F).

SDC4 showed no immunostaining in the stroma of normal tissue (Figure 2J). SDC4 showed moderate epithelial immunostaining in the PIN stage (Figure 2K) from the Pten mouse. Epithelial and stromal immunostaining of SDC4 was present in the advanced stage in the Pten mouse (Figure 2L). There was no immunostaining in the PIN or advanced tumor stage for SDC4 in the p53/Rb mouse (Figure 3G,H). SDCBP showed poor immunostaining in the normal prostatic epithelium (Figure 2M). However, SDCBP immunostaining was moderate in tumor cells in the PIN and advanced stages of the tumor in the Pten mouse (Figure 2N,O). In the p53/Rb mouse, strong expression of SDCBP was observed only in the PIN stage, and no reaction was observed in the advanced tumor stage (Figure 3I,J). Furthermore, no significant differences were observed in the immunostaining of target proteins between the different prostate lobes.

### 2.3. Immunohistochemical Analysis of SDC Family Members and SDCBP in Human Prostate Tissues

**Figure 4:**
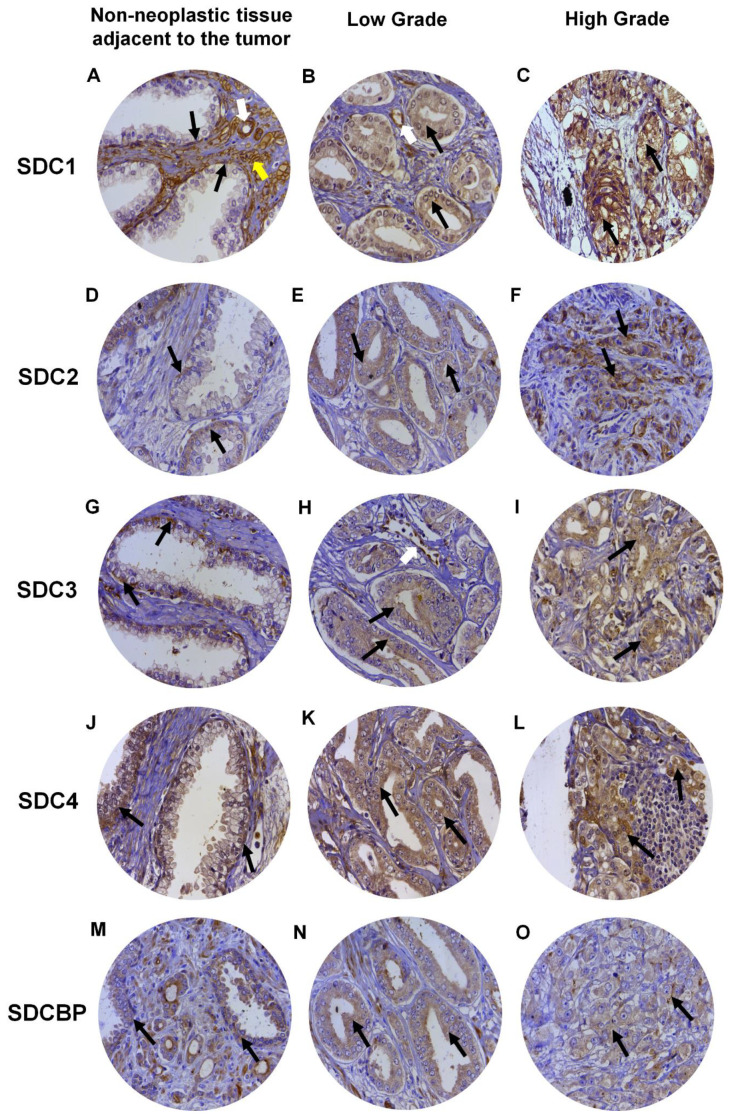
Representative images of immunohistochemical staining for SDC1, -2, -3, and -4, and SDCBP in adjacent non-neoplastic tissue (**I**), low-grade Gleason (Gleason grade 3), and high-grade Gleason (Gleason grades 4 or 5) from TMAs of human prostate samples. Images (**A**–**C**) show SDC1 staining. Images (**D**–**F**) show SDC2 staining. (**G**–**I**) show SDC3 staining. The (**J**–**L**) images show SDC4 staining. (**M**–**O**) images show SDCBP staining. Non-neoplastic tissue: (**A**,**D**,**G**,**J**,**M**). Low Grade: (**B**,**E**,**H**,**I**,**K**). High Grade: (**C**,**F**,**I**,**L**,**O**). Black arrows indicate positively stained cells. White arrows: blood vessels. Yellow arrow: smooth muscle cells. Final magnification: ×400.

Expression of SDC1 in normal prostate tissues (non-neoplastic tissue adjacent to the tumor), when positive, showed strong immunostaining in the basal cells of the epithelium and around the smooth muscle cells of the stroma and blood vessels (Figure 4A). In adenocarcinomas, SDC1 immunostaining was not present in some patient samples; however, it showed positive immunostaining in epithelial cells, in both Gleason 3 (low grade) and Gleason 4–5 adenocarcinomas (high grade) (Figure 4B,C). SDC2, SDC3 and SDC4 expression in normal tissue, when positive, were present at the basolateral membranes of luminal epithelial cells and in basal cells (Figure 4D,G,J). In adenocarcinomas, positive immunostaining for SDC2 was detected, with moderate immunostaining present throughout the cytoplasm of neoplastic epithelial cells of patients with Gleason 3 tumors (Figure 4E) and strong cytoplasmic and pericellular immunostaining in Gleason 4–5 tumors (Figure 4F). No immunostaining for SDC2 was observed in the glandular stroma.

In adenocarcinomas, when positive, SDC3 showed moderate immunostaining throughout the cytoplasm of neoplastic epithelial cells of patients with Gleason 3 tumors (Figure 4H) and weak cytoplasmic and pericellular immunostaining in Gleason 4–5 tumors (Figure 4I). Immunostaining of SDC3 was observed around blood vessels. Positive immunostaining was detected for SDC4 in adenocarcinomas, where strong immunostaining was observed throughout the cytoplasm of neoplastic epithelial cells of patients with Gleason 3 (Figure 4K) and Gleason 4–5 tumors (Figure 4L). Immunostaining of SDC4 was observed in the glandular stroma and cells of the immune system.

SDCBP expression in normal tissue, when positive, was present in the cytoplasm of luminal and basal epithelial cells (Figure 4M). Positive immunostaining was detected for SDCBP in adenocarcinomas. SDCBP presented moderate immunostaining throughout the cytoplasm of neoplastic epithelial cells of patients with Gleason 3 tumors (Figure 4N) and weak cytoplasmic and pericellular immunostaining in Gleason 4–5 tumors (Figure 4O). Immunostaining for SDCBP was present in the epithelial stroma of normal and tumor tissues. Figure S1 summarizes the number of patients with positive and negative immunostaining for each marker and their distribution in the ISUP (International Society of Urological Pathology) prognostic category.

### 2.4. Gleason Score Correlation and Survival Curves

After the immunohistochemistry analysis, 106, 105, 105, 101, and 102 sections remained in the slides to be analyzed for SDC1, SDC2, SDC3, SDC4, and SDCBP, respectively, from an initial 119 patient samples. Table 1 shows the number of patient samples with positive and negative immunostaining results for each marker and their distribution in the Combined Gleason Score. From all five makers, only cytoplasmic expression of SDC3 proved to be associated with an increased Gleason score and higher tumor stage (*p* = 0.0053). We also investigated the immunostaining patterns in the stromal components for SDCs and SDCBP, and besides some specificities, no prognostic value for these specificities was observed (Table S1 and Figure 4).

**Figure 5:**
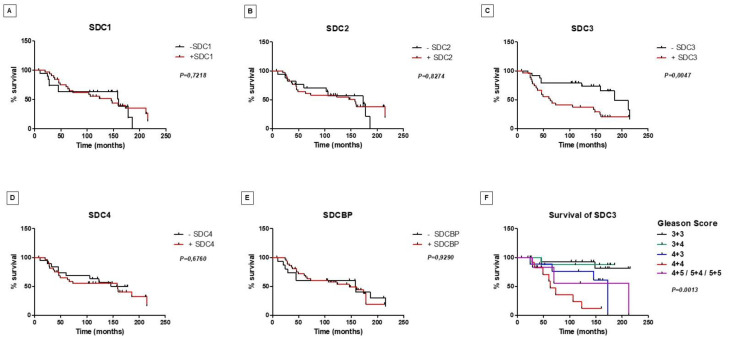
Prostate-specific survival of patients with prostate cancer regarding syndecans and syntenin-positive and -negative tissue protein immunostaining. Kaplan–Meier curves for survival to SDC1 (**A**), SDC2 (**B**), SDC3 (**C**), SDC4 (**D**), and SDCBP (**E**). Note the reduced survival of patients with positive immunostaining for SDC3, *p* = 0.0047. (**F**) Stratification of patients positive for SDC3 by Gleason score. SDC3 positive staining revealed lowest prostate-specific survival for patients with 4 + 4 Gleason score (*p* = 0.0013).

Along with the results obtained for the p53/pRb mice, these results suggested that SDC3 is associated with tumor progression. Prostate-specific survival did not show a significant difference between patients who had PCa samples with positive or negative immunostaining for SDC1, SDC2, SDC4, and SDCBP (Figure 5A–F). However, as shown in Figure 5C, patients with positive immunostaining for SDC3 showed a shorter prostate-specific survival than those with negative immunostaining (*p* = 0.0047). Among patients positive for SDC3, patients with Gleason score 4 + 4 had the lowest survival (*p* = 0.0013) (Figure 5F).

### 2.5. Prognostic Value by Time of Biochemical Recurrence Analysis Using Gene Expression Patterns in Public Datasets

**Figure 6:**
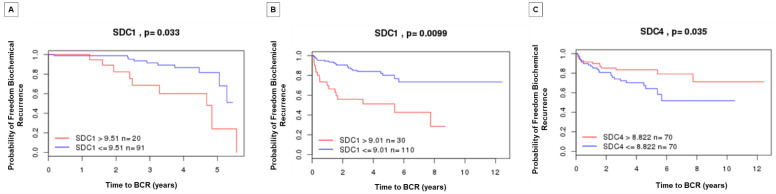
Kaplan–Meier curves displaying the probability of freedom from biochemical recurrence of PCa with (**red**) or without (**blue**) *SDC1* and *SDC4* overexpression. Analyzed by the Cambridge Carcinoma of the Prostate App (camcAPP dataset) [37] from an integrative study. (**A**) Kaplan–Meier curve with the probability of freedom from biochemical recurrence of PCa with (**red**) or without (**blue**) *SDC1* overexpression from the Cambridge study [38]. The difference is statistically significant, *p* = 0.033. (**B**) Kaplan–Meier curve with the probability of freedom from biochemical recurrence of PCa with (**red**) or without (**blue**). *SDC1* overexpression from the Memorial Sloan-Kettering Cancer Center (MSKCC) study [39]. The difference is statistically significant, *p *= 0.0099. (**C**) Kaplan–Meier curve with the probability of freedom from biochemical recurrence of PCa with (**red**) or without (**blue**) *SDC4* overexpression from the MSKCC study [39]. The difference is statistically significant, *p* = 0.035.

**Figure 7:**
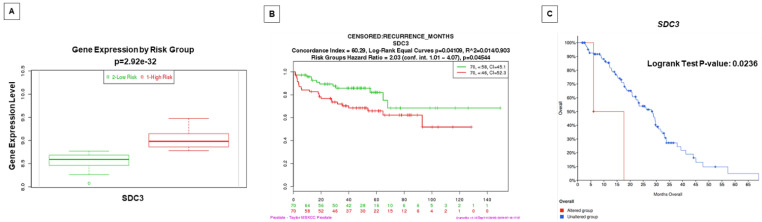
Gene expression of *SDC3* in patients with PCa is associated with high risk/worse prognosis and lower disease-/progression-free survival. (**A**) The level of *SDC3* gene expression (median) in PCa patients with low risk (**green**) and patients with high risk (**red**). Data and analyses were cataloged using the Survexpress database [40] from an MSKCC prostate study [39]. The difference between boxplots is statistically significant with *p* = 2.92 × 10^−32^. (**B**) Kaplan–Meier curve displaying risk group survival of PCa patients with low risk (**green**) and high risk (**red**) of *SDC3* alterations, cataloged using the Survexpress database [40] from the MSKCC prostate study [39]. Curves are significantly different, with *p *= 0.04544. (**C**) Overall patient survival status in Metastatic Prostate Adenocarcinoma patients with SDC3 gene-altered (**red**) and -unaltered (**blue**) groups. Data and analyses were cataloged using the cBioPortal database [41,42] (Metastatic Prostate Adenocarcinoma—SU2C/PCF Dream Team) [43].

Gene expression patterns and prognosis of patients with PCa from *SDC1*, *SDC4*, and *SDCBP* were analyzed by comparison with five published datasets (TCGA, MSKCC, Cambridge, Stockholm, and SU2C/PCF Dream Team). In these analyses, overexpression of the *SDC1* gene was associated with reduced time of biochemical recurrence in two datasets (Cambridge and MSKCC) (Figure 6A,B). *SDC3* gene overexpression showed prognostic value for reduced biochemical recurrence in the MSKCC dataset and overall survival for metastatic disease in the SU2C/PCF Dream Team dataset (Figure 7). *SDC4* overexpression was associated with a good prognosis for biochemical recurrence in the MSKCC dataset (Figure 6C). A non-significant result in prognostic value from the different queried studies was found (Figure S2).

## 3. Discussion

The expression of proteoglycans of the syndecan family has been associated with prognosis and treatment response in a wide range of cancers, including hematological malignancies and solid tumors [44,45,46]. However, the role of syndecan family members in the prognosis of PCa is still controversial [29,47]. This study used two transgenic mouse models of PCa and tumor samples from PCa patients with clinical data to evaluate the tissue expression pattern of proteoglycans of the syndecan family and SDCBP and its behavior during the process of tumor progression, patient biochemical recurrence, and survival. Our study is the first to evaluate all four syndecans simultaneously in two mouse models to further explore in vivo the role of SDCs in prostate cancer progression.

Most previous studies examining the expression of syndecans in tumors and prostatic tissue focused on SDC1 [32,48]. Our research found higher expression and immunostaining of *Sdc1*/SDC1 in the Pten mouse and lower expression in a neuroendocrine tumor in the p53/Rb mouse. This result contrasts with the results shown by Shimada et al. 2013 [49], who reported that SDC1 contributes to tumor progression by stabilizing cancer-initiating cells, favoring the growth and incidence of metastases in the TRAMP mouse, which is also characterized by the presence of neuroendocrine tumors [49]. In our cohort, tissue microarray analysis of SDC1 protein tissue expression did not reveal an association between immunostaining and patient prognosis and survival. However, a significant association between SDC1 immunostaining and worse prognosis was found for patients in two previously published datasets [38,39]. The prognostic value of SDC1 immunostaining in PCa has also been reported by others [32,50,51]. Recently, SDC1 has also been reported as a serum marker for patients who are non-responsive to docetaxel therapy [33]. These studies strongly support the prognostic significance of SDC1 in PCa patient survival.

Although two previous studies have associated *SDC2* expression with a worse prognosis in PCa patients [36,51], in our study, no association was found between *SDC2* gene expression levels or protein tissue expression and a favorable or unfavorable prognosis for PCa patients in any of the investigated datasets. Thus, the prognostic significance of *SDC2* expression in PCa remains unclear. Our study showed that *Sdc3* is highly expressed at both the mRNA and protein levels in advanced p53/Rb mouse tumors. These tumors are highly invasive and metastatic [52,53], and are negative for the androgen receptor (result not shown).

*SDC3* is one of the least-studied syndecans in PCa [54]. Recently, *SDC3* expression was associated with perineural invasion in pancreatic cancer [55,56]. Our survival analysis revealed a worse prognosis for the group of patients with positive SDC3 immunostaining. In addition, we also found a poor prognosis for patients with a high expression of SDC3 in primary and metastatic tumors from other studies. To the best of our knowledge, this is the first study to demonstrate a predictive value of SDC3 in PCa with a strong association with a high Gleason score. Future studies are needed to better understand the role of SDC3 in PCa progression.

SDC4 showed significantly reduced expression in the advanced tumor stage of the p53/Rb mouse. The same results were observed in non-seminomatous germ cell tumors, in which immunostaining for SDC4 in advanced stages was reduced [57]. The authors attributed the loss of SDC4 staining on the surface of tumor cells to the aggressiveness and stage of tumor progression, with higher expression of *SDC4* in less-aggressive tumors [57]. In our patient group, no association was observed between the pattern of SDC4 immunostaining and patient survival. However, in the MSKCC dataset, we found a significantly worse prognosis of biochemical recurrence for lower *SDC4* gene expression patients. The underlying mechanisms and functional consequences of SDC4 in PCa will require further investigation.

Studies have suggested that SDCBP, also known as “Melanoma differentiation-associated gene-9” (MDA-9), participates in invasion and metastasis in several cancers [58,59], albeit mainly in melanomas [30]. Our study found no significant change in the gene expression levels of *Sdcbp* in mouse tumors. There was no association between *SDCBP* expression and patient prognosis in human prostate cancer datasets. Recently, *Sdcbp* has been documented as up-regulated in Hi-Myc mice adenocarcinomas and stages II and III of prostate tumors, compared with the adjacent normal tissue. By physically interacting with IGF-1R, SDCBP activates STAT3 and thus regulates prostate cancer pathogenesis [60,61]. Moreover, it has been demonstrated that PDZ1, an SDCBP target-specific small-molecule inhibitor, displays therapeutic potential for prostate cancer and potentially other cancers expressing elevated levels of SDCBP [62]. These results suggest that, although gene and protein expression levels of SDCBP do not exhibit a prognostic value, patients harboring a higher Gleason score may benefit from a therapeutic approach involving SDCBP inhibitors or down-regulation as proposed by others [63], particularly those with elevated levels of SDC expression.

Gleason’s score remains the best method for PCa staging. However, it is poorly predictive of disease progression, recurrence, and metastasis. Therefore, additional molecular markers are still necessary. We believe that the present characterization of syndecans and syntenin expression patterns might contribute to a more elaborate prognostic of PCa progression. These results also highlight the importance of an integrative approach with protein tissue expression to clarify possible contributions of stromal cells in the tumoral mRNA pool from lysed tissues for prognostic evaluation. Our work also presented two mouse models to further explore, in vivo, the role of SDCs in prostate cancer progression, as well as preclinical studies of SDCs pathways as therapeutic targets.

## 4. Materials and Methods

### 4.1. Gene Expression Analysis of Sdc1-4 and Sdcbp in Two Genetically Engineered Mouse Models (GEMM) of PCa: Pten and p53/Rb Conditional Knockouts

We used RNAseq data and prostate samples from different stages of tumor development and progression in two established GEMM of prostate cancer: the *Pb-Cre4/Pten^f/f^* (Pten mouse), which develops castration-sensitive, invasive, but rarely metastasizing cancer [64,65], and the *Pb-Cre4/Trp53^f/f^-;Rb1^f/f^* (p53/Rb mouse), which develops metastatic castration-resistant prostate cancer [66]. These mice show stages of tumor progression similar to those of human PCa, such as prostatic intraepithelial neoplasia (PIN), micro-invasive and invasive well-differentiated adenocarcinoma (medium-stage tumors), and fully invasive poorly differentiated adenocarcinoma (advanced-stage tumors). Moreover, deletions and mutations of the tumor suppressors *PTEN*, *TP53*, and *RB1* are among the most common genomic alterations in human prostate cancer. They have been consistently associated with more aggressive disease features and worse prognosis [67,68]. Although there are other interesting GEMMs for PCa, few studies have combined the stages of tumor progression with all prostatic lobes (anterior, ventral, lateral, and dorsal prostate) in a deep RNA sequencing experiment. Additional details about these conditional knockout mice, histopathological analysis, and transcriptome data have been previously described [52,53].

We accessed the RNA sequencing data samples of all four prostatic lobes through the NCBI Gene Expression Omnibus (GEO, https://www.ncbi.nlm.nih.gov/geo/) (accessed on 15 July 2021) platform, reference number GSE94574. Briefly, 93 samples were submitted to RNAseq analysis, including 20 wildtype prostatic lobes, 32 PIN-stage tumors, 20 medium-stage tumors, and 21 advanced-stage tumors. At least four samples were submitted to RNAseq analysis for each prostatic lobe and pathological condition for each mouse. Details of the RNAseq procedure have been published before [53,67]. Histopathological description of each tumor stage used for total RNA extraction is presented in the Supplementary File (Figure S3).

### 4.2. Immunohistochemistry (IHC) Staining in Mouse Sample Analysis

We obtained paraffin blocks of all prostatic lobes containing WT and tumoral samples from GEMM from David Neal’s Uro-Oncology Group at CRUK Cambridge Institute (University of Cambridge, UK). At least 10 different urogenital complex paraffin blocks from the wild type and 20 from both knockout mice were sectioned. Histological sections of the prostate at various stages of development and progression were deparaffinized, hydrated, and washed in PBS (0.1 M, pH 7.4). Antigen retrieval was performed using 10 mM citrate buffer pH 6.0 for 35 min in a Dako Cytomatica pressure cooker. Subsequently, slices were submitted to endogenous peroxidase blockade with 3% H_2_O_2_ solution in methanol for 10 min, protein–protein interaction block with 3% BSA in PBS, and overnight incubation at 4 °C with primary antibodies against SDC-1 (AB128936), -2 (AB79978), -3 (AB191308), -4 (AB24511), or SDCBP (AB-19903), diluted 1:100 in 1% BSA solution in PBS. The five antibodies were purchased from Abcam (Cambridge, UK). After washing with PBS, the sections were exposed to the peroxidase-conjugated secondary antibody, developed using diaminobenzidine as a chromogen, and counterstained with hematoxylin. We analyzed slides using a Leica DM2500 microscope and acquired images with a Leica DMC2900 camera and Leica Qwin image analysis software version 3.1.

### 4.3. Patient Tissue Sample Microarray

Prostate samples were obtained from 119 patients with prostate carcinoma, with a median age of 64 years (range 46–74), selected from a cohort of patients who underwent radical prostatectomy at the Botucatu Medical School Hospital (HC/FMB) as primary therapy (without hormone therapy or radiotherapy) for clinically localized PCa between 1989 and 2000. TMAs were constructed as previously reported [69,70]. A description of the clinical data of the patients used in the preparation of the TMA (Figure S4), such as Gleason score, survival time, and patient outcome, is presented in Table S2. The Medical Ethics Committee of FMB/UNESP approved this study (Protocol N°. 3888/2011).

Although there is no universal method of sampling PCa tissue for immunohistochemical staining using standard slides or TMAs, the histological characteristics of the sample areas were representative of the final Gleason score for each case. A TMA was constructed using representative samples of adjacent non-neoplastic prostate tissue and prostate cancer. We used two tissue cores of 1 mm diameter for each sample. PCa samples from patients, organized in a tissue microarray (TMA), underwent immunohistochemical reaction, using the same procedure as described for mouse samples. Immunohistochemical reactions were evaluated and quantified according to the staining intensity score: negative or weak staining scored 0, and tumors with medium and strong intensity scored 1.

Analysis was performed by two independent observers, in a blinded manner, without access to the clinical data of the patients and to the target of the antibody, and a joint review resolved any difference. We associated the findings with the Gleason score and the patient’s prostate-specific survival. We generated Kaplan–Meier plots using positive (score 1) and negative (score 0) staining as cut-offs. In addition, we produced a survival curve stratified by Gleason score of the patients with positive staining for markers with statistically significant prognostic value (Figure 6F). The distribution of patients with positive and negative staining for each marker and by ISUP prognostic category is presented in Figure S1. Patients who died from the disease were used for prostate-specific survival curve analysis. The other cases died due to different reasons or had no information in their medical record and were therefore discarded from this analysis. We constructed the Kaplan–Meier survival curve in association with positive and negative expression of the five different markers.

### 4.4. Prognostic Value Analysis in Public Datasets

Finally, we investigated the expression pattern of the SDC1-4 and SDCBP genes in published PCa datasets. The gene expression pattern was analyzed using the SurvExpress database [40], and the Cambridge Carcinoma of the Prostate App (CamcAPP) database developed by the Cancer Research UK Cambridge Institute [37] (https://bioinformatics.cruk.cam.ac.uk/apps/camcAPP/) (accessed on 15 July 2021); and cBioPortal for Cancer Genomics database [41,42] (https://www.cbioportal.org/) (accessed on 15 July 2021), to determine the association of gene alterations with patient clinical data, such as risk/prognosis and survival rates.

Gene expression was associated with poor outcomes (decreased relapse-free survival and gene expression level in the worst risk group) in published PCa datasets—Cambridge study [38], MSKCC study [39], and Metastatic Prostate Adenocarcinoma—SU2C/PCF Dream Team [43].

### 4.5. Statistical Analysis

A one-way ANOVA with Dunnett’s multiple comparison post-test (wildtype vs. each group) was used for gene expression analysis. A chi-square contingency test was used for the association analysis of Gleason score (Table 1) and immunostaining markers. The survival curve was constructed using the Kaplan–Meier method and compared using the log-rank (Mantel–Cox) test. Differences were considered statistically significant when *p* ≤ 0.05. Statistical analyses were performed using the GraphPad Prism program v. 5.0 (San Diego, CA, USA).

## 5. Conclusions

Syndecans and syntenin expression patterns at the various stages of the prostate tumor vary according to the genetic heterogeneity of the tumors. Our results suggest that SDC4 expression correlates with indolent tumors and a better prognosis. In comparison, SDC1 and SDC3 are prevalent in more aggressive tumors and could be used as biomarkers of worse prognosis for PCa patients. Additional preclinical and clinical studies are required to further validate the roles of SDC1, 3, and 4 as valuable biomarkers for risk-stratification of localized PCa.

## Figures and Tables

**Table 1:** Association between the Syndecans (SDC) 1–4 and Syntenin-1 immunostaining results and prostate cancer Gleason score ^1^.

Protein	Gleason Score				*Chi-Square* *p*-Value
	<7 (*n* = 46–50)	=7 (*n* = 18–19)	>7 (*n* = 36–37)	Percentage (%)	
**SDC1**					
Positive	34	14	29	72.64	0.5585
Negative	16	5	8	27.36	
**SDC2**					
Positive	23	12	23	55.24	0.2774
Negative	26	7	14	44.76	
**SDC3**					
Positive	9	6	19	32.38	0.0053
Negative	40	13	18	67.62	
**SDC4**					
Positive	27	13	29	68.32	0.1076
Negative	19	6	7	31.68	
**SDCBP**					
Positive	40	14	29	81.37	0.8646
Negative	8	4	7	18.63	

^1^ The number of samples does not always add up to 115 in different markers because some losses occurred during the IHC procedures.

## Data Availability

Data presented in this article is available from the corresponding author upon reasonable request.

## Supplementary Materials

The following are available online at https://www.mdpi.com/article/10.3390/ijms22168669/s1.
