# Supplementary material for: Syndecan Family Gene and Protein Expression and Their Prognostic Values for Prostate Cancer
Source: Int J Mol Sci. 2021 Aug 12;22(16):8669. doi: 10.3390/ijms22168669 (PMC8395474; doi:10.3390/ijms22168669)
Supplement: Supplementary file 1 [file ijms-22-08669-s001.zip › ijms-1314678-supplementary.pdf]

Supplementary Files

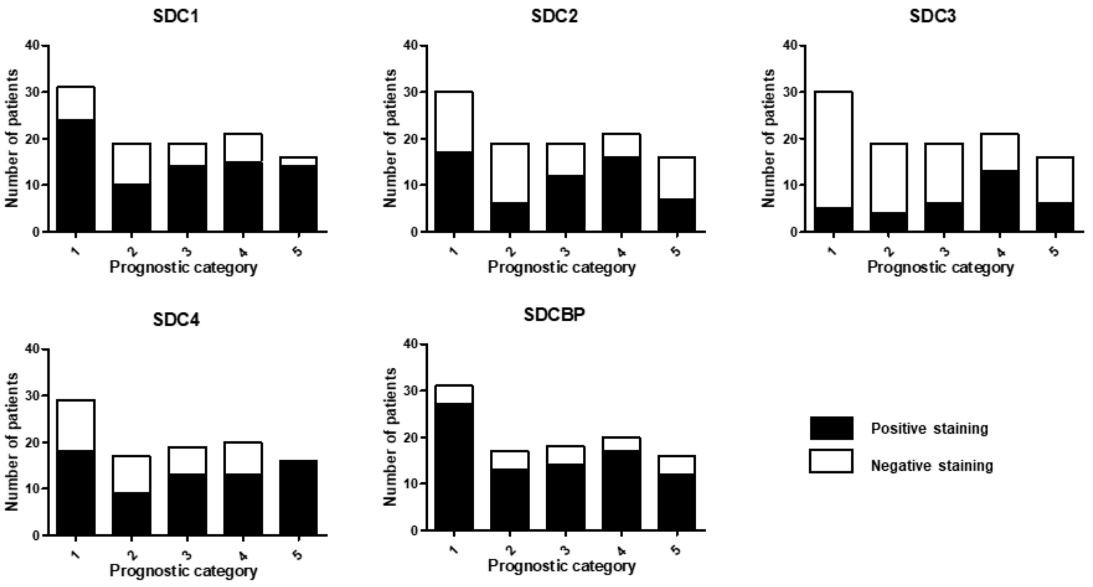

Figure S1: Number of patients per ISUP (International Society of Urological Pathology) prognosis associated with positive and negative staining for SDC1, -2, -3, and -4 and SDCBP. Representative graphs show the association between SDC family members and SDCBP protein expression (by immunohistochemistry in human prostate TMAs) and prognosis according to ISUP. White bars represent patients with negative immunostaining, and black bars represent patients with positive immunostaining.

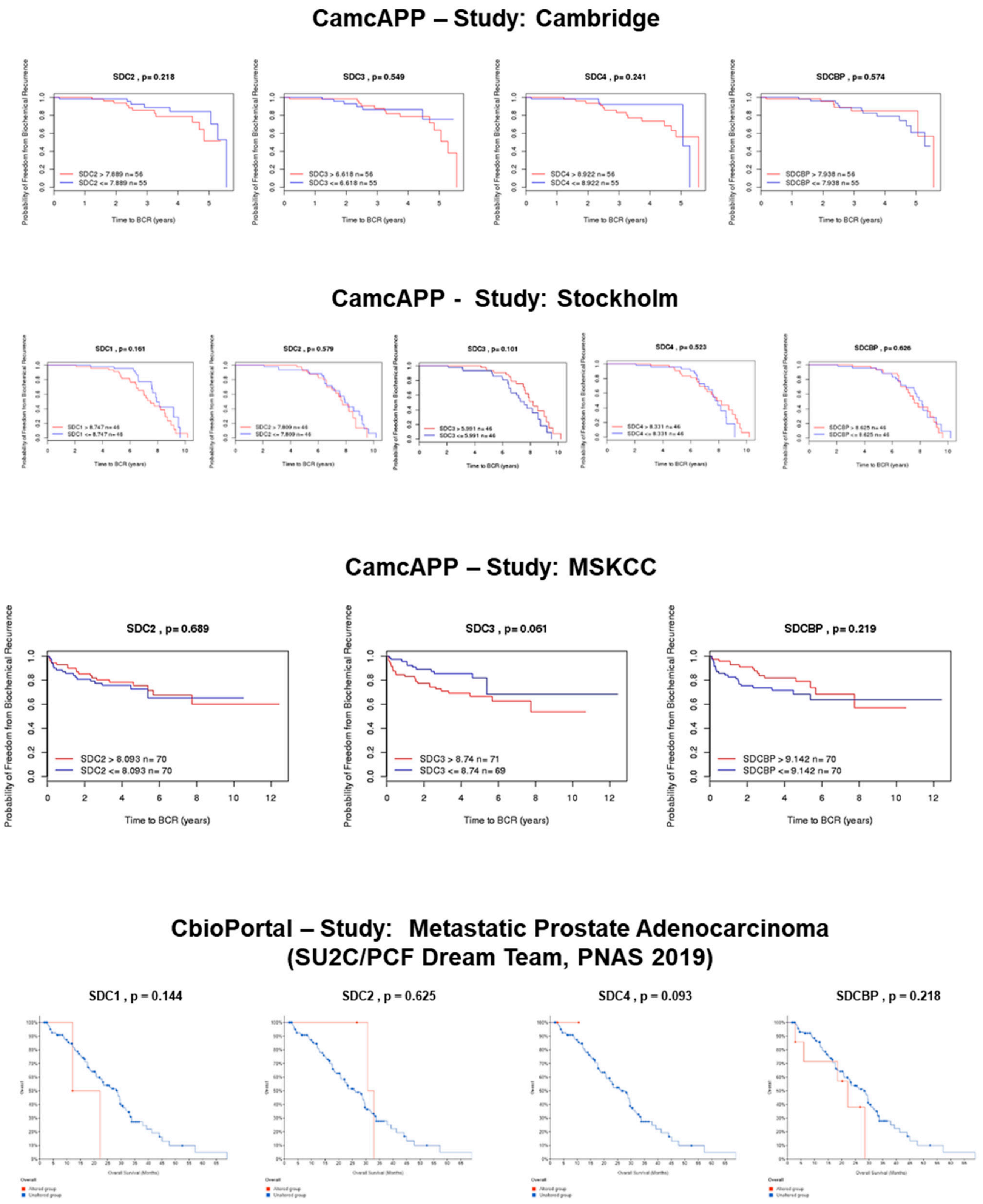

Figure S2: Kaplan Mayer curves displaying the probability of freedom from Biochemical recurrence of prostate cancer. Results with not significant prognostic value for SDC1-4 and SDCBP gene expression levels from Cambridge [36,37], Stockholm [36,37], MSKCC [38] studies.

Prostatic lobe histopathology of genetically engineered mouse models (GEMM)

Histopathology was performed on the two genetically engineered mouse models (GEMM) of PCa utilized in the gene expression analysis, and Figure S3 shows immunostaining for SDCs. The two GEMM of PCa showed different histopathological characteristics and stages of tumor progression.

Normal prostatic lobes or non-neoplastic tissue (Wild type) exhibited acini lined by single cubic or columnar epithelial secretory cells, surrounded by a reduced stroma (Figures S3A–D). In the *Pb-Cre4/Pten$^{f/f}$* GEMM (Pten mouse), the first stage was observed as prostatic intraepithelial neoplastic (PIN) lesions, which were present in the anterior (AP), ventral (VP), dorsal (DP), and lateral (LP) prostate lobes. PIN was characterized by stratified structures of neoplastic epithelial cells but confined by the basement membrane in the luminal space of the acini. PIN lesions in mice may be classified as low grade (PIN I and II) or high grade (PIN III and IV). After four months of age, most sections showed lesions classified as PIN III and IV (high grade), with a cribriform pattern, in the prostatic lobes (Figures S3E–H). These PIN lesions progress to medium-stage tumors (MedTumor), where most of the glandular structures present high-grade PIN with microinvasion and prominent reactive stroma (Figures S3I–L). After 12 months of age, larger heterogeneous areas of fully invasive, both well- and poorly differentiated adenocarcinoma associated with reactive stroma were found (Figures S3M–P) and designated as advanced-stage tumors (AdTumor). In this study, none of the Pten mice developed metastasis.

The *Pb-Cre4/Trp53$^{f/f}$-;Rb1$^{f/f}$* GEMM (p53/Rb mouse) exhibited low-grade PIN lesions (PIN I and PIN II, low grade) at focal points of the prostatic epithelium in all prostatic lobes (Figures S3Q–T). These lesions did not progress to more advanced stages. However, in the proximal regions of the prostatic ducts, a large mass of undifferentiated neuroendocrine (NEPC) and invasive adenocarcinoma (Figure S3U) was observed. Cells in NEPC adenocarcinomas had minimal cytoplasm and hyperchromatic cell nuclei (Figure S3V).

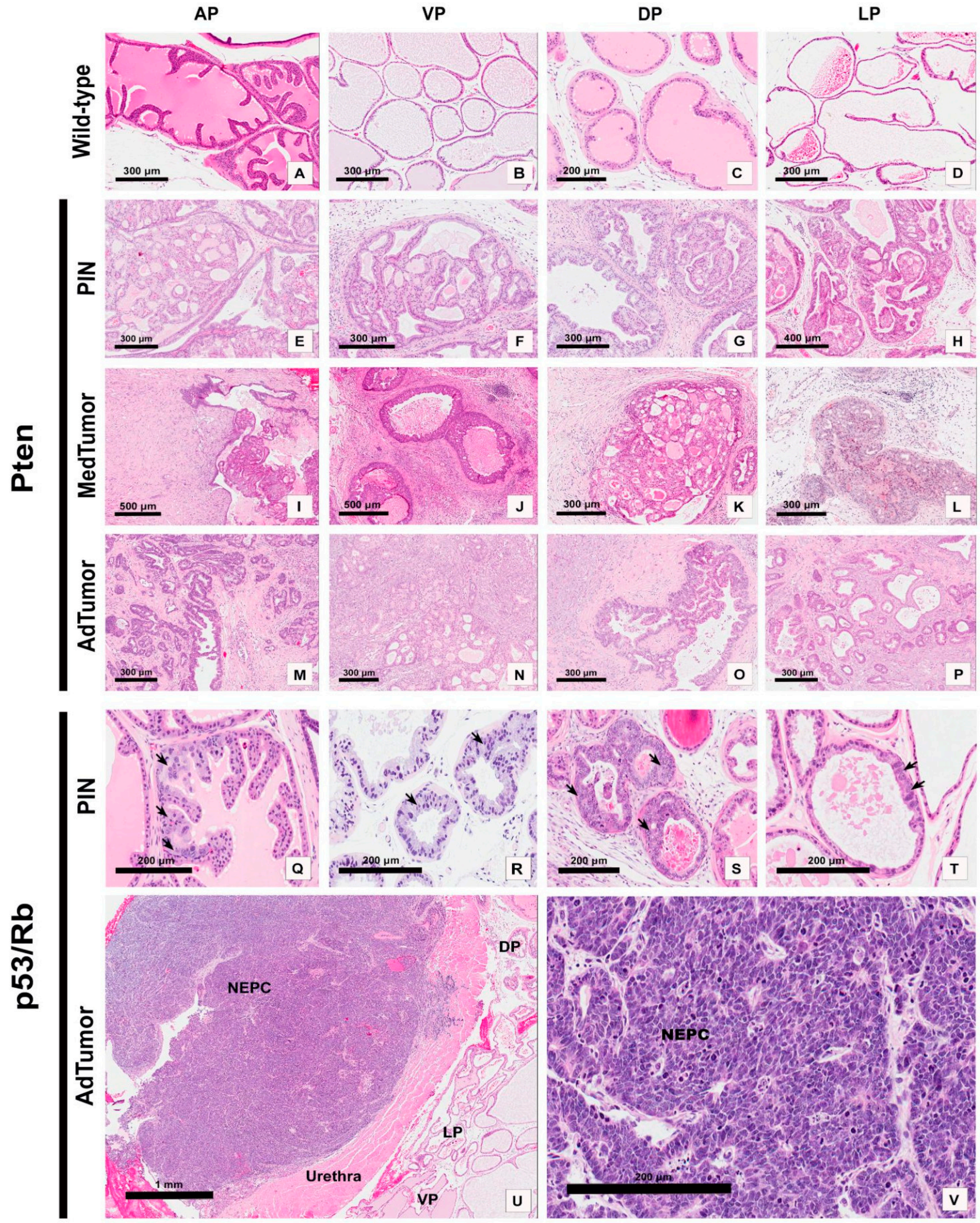

Figure S3. Representative histopathological images of normal (*Pb-Cre4-negative* – wild type) and tumoral prostatic lobes from *Pb-Cre4/Pten*$^{f/f}$ (Pten) and *Pb-Cre4/Trp53*$^{f/f}$*-;Rb1*$^{f/f}$ (p53/Rb) genetically engineered mouse models. A–D: Wildtype prostatic lobes. E–P: Three different stages of tumor progression were observed in the prostate tumor in the Pten mouse. E–H: PIN – Prostatic Intraepithelial Neoplasia. I–L: MedTumor – Middle stage tumor, micro-invasive adenocarcinomas; M–P: AdTumor – Tumor in a more advanced stage, invasive adenocarcinomas. Q–V: Two different stages of progression observed in prostate tumors from p53/Rb mouse. Q–T: PIN – Prostatic Intraepithelial Neoplasia. U–V: Prostatic tumor originating from the proximal region of the urethra, showing a typical pattern of undifferentiated neuroendocrine tumors. AP: Anterior Prostate; VP: Ventral Prostate; LP: Lateral Prostate; DP: Dorsal Prostate; NEPC: Neuroendocrine Prostate Cancer. Scale bars: A-T and V = 200 µm; U = 1 mm.

Figure S4. Tissue microarray slices from patient's prostate tumors used for each marker.

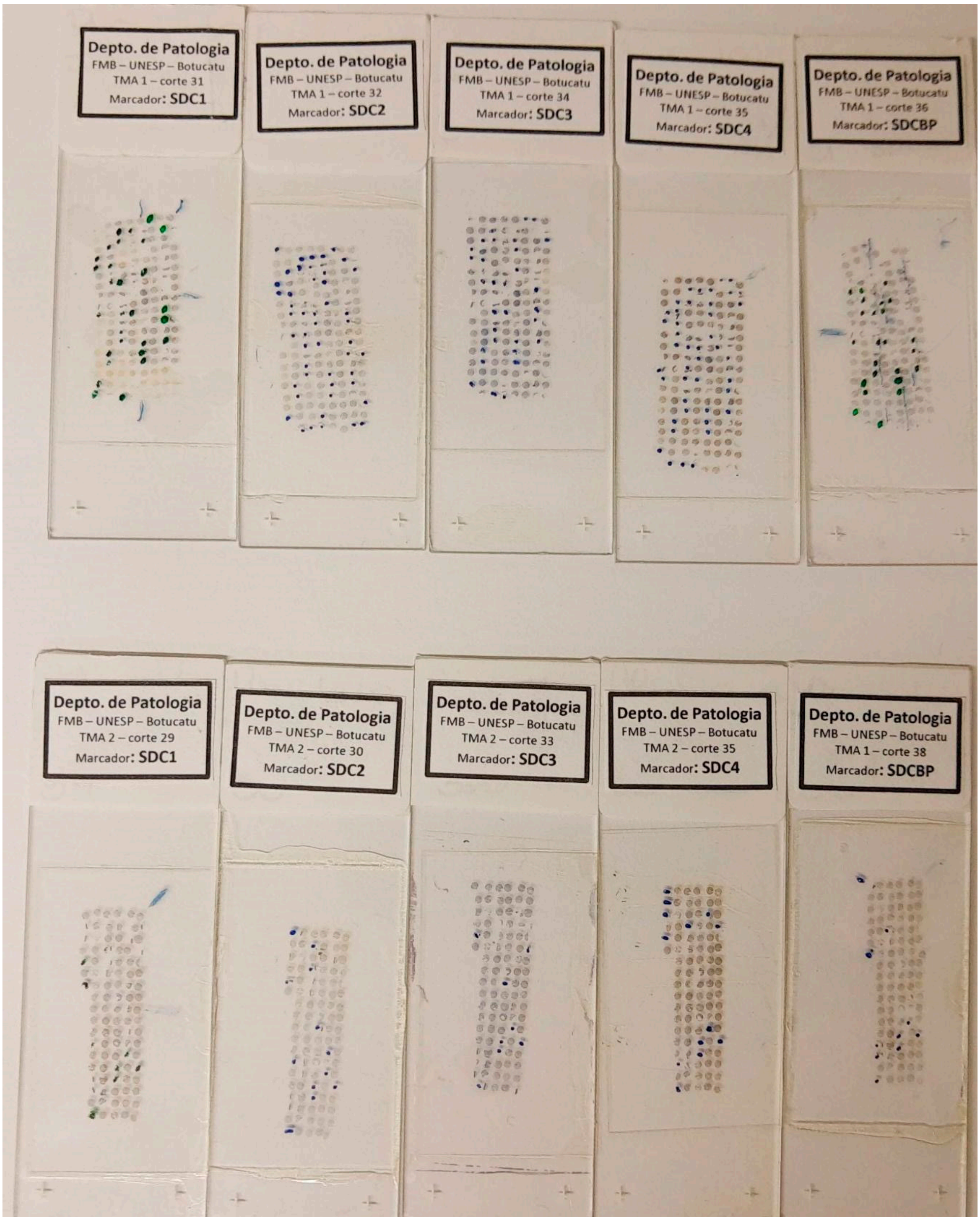

Table S1. Association between the syndecans (SDC) 1-4 and syntenin-1 immunostaining results and prostate cancer stromal components.

Protein		Stromal Components				*Chi-square*
		Connective tissue	Blood vessels	Nerves	Smooth muscle cells	P-value
**SDC1 (n = 107)**						
	Positive	6	92	0	41	**< 0.0001**
	Negative	101	15	107	65	
**SDC2 (n = 102)**						
	Positive	5	3	3	14	**0.0028**
	Negative	97	99	99	88	
**SDC3 (n = 111)**						
	Positive	0	95	9	0	**< 0.0001**
	Negative	111	16	102	111	
**SDC4 (n = 115)**						
	Positive	66	0	0	0	**< 0.0001**
	Negative	49	115	115	115	
**SDCBP (n = 106)**						
	Positive	17	1	0	25	**< 0.0001**
	Negative	89	105	106	81	

Numbers of samples do not always add up to 115 in different markers, because some losses occurred in the IHC procedures.

Table S2: Patient's clinical data used in the preparation of the TMA, such as Gleason Score reviewed, prognostic category, survival time, and patient outcome.

TMA Number	Gleason	Prognostic Category (ISUP)	Survival time (months)	Outcome / Final Result	SDC1	SDC2	SDC3	SDC4	SDCBP
PR1	4+4	4		No information		1	1	1	1
PR2	4+4	4		No information	1	1	1	0	1
PR3	4+4	4	123	Death by disease	1	1	1	0	1
PR4	4+4	4	123	Death by disease	1	1	1	1	1
PR5	4+4	4	63	Death by disease	1	1	1	1	1
PR6	4+4	4	63	Death by disease	1	1	1	1	1
PR7					1	1	0	1	0
PR8					1	1	0	1	1
PR9					1	1	1	1	1
PR10	4+4	4	28	Death by disease	0	0	0	1	1

PR11	4+5	5		No information	1	1	0	1	1
PR12	4+5	5		No information	1	1	0	1	1
PR13	4+4	4	32	Death by disease	1	1	1	0	1
PR14	3+4	2		Loss of follow-up					
PR15	5+4	5	36	Death by disease	1	0	0	1	1
PR16	4+4	4		No information	1	1	0	1	1
PR17	5+4	5		No information	1	1	0	1	0
PR18	4+3	3		No information	1		0	1	
PR19	4+4	4	26	Death by disease	0	1	0	1	1
PR20	4+4	4	160	Death by disease	0	1	0	0	0
PR21	3+3	1		No information	1	1	1	0	1
PR22	4+4	4	49	Death by disease	1	0	1	0	1
PR23	4+3	3		Loss of follow-up	1	0	0	0	1
PR24	5+5	5		No information	1	1	0	1	1
PR25	3+3	1	215	Death by disease	1	0	0	1	1
PR26	5+5	5		No information	1	0	0	1	1
PR27	3+4	2		Loss of follow-up	0	1	0	1	0
PR28	3+3	1		No information	0				1
PR29	4+4	4		No information	0	1	1	0	
PR30	3+4	2		Loss of follow-up	1	1	1	0	
PR31	5+5	5	120	Death with disease (another cause)	1	0	0	0	1
PR32	4+3	3	146	Death by disease	1	1	1	1	1
PR33	3+3	1		Loss of follow-up	1	1	0	1	1
PR34	3+4	2		Loss of follow-up					
PR35	3+3	1	159	Death by disease	1	1	0	0	0
PR36	3+4	2	186	Death by disease	0	0	0	1	1
PR37	4+4	4	24	Death by disease	0				
PR38	3+3	1		Loss of follow-up	1	1	0	0	1
PR39	3+4	2		No information	1	1	1	0	
PR40	4+4	4		Loss of follow-up	1	0	0	0	1
PR41	3+4	2		Loss of follow-up	1	1	1	1	1
PR42	3+3	1	148	Death without disease	1	1	1	1	1
PR43	3+3	1		No information	1	0	0	1	1
PR44	3+4	2		Loss of follow-up	1	0	0	1	1
PR45	3+3	1	212	Death without disease	0	1	0	1	0
PR46	4+3	3		Loss of follow-up		1			
PR47	3+3	1	146	Death without disease	1	1	0	1	1
PR48	3+4	2		Death with disease (another cause)	1	0	0	0	1
PR49	4+4	4	46	Death by disease	1	1	0	1	1
PR50	4+3	3	8	Death with disease (another cause)	1	1	0	1	1

PR51	5+4	5	105	Death with disease (another cause)					
PR52	4+4	4	20	Death by disease	1	1	0	1	0
PR53	4+3	3		Loss of follow-up	1	0	0	1	0
PR54	4+3	3		No information	0	0	1	1	0
PR55	4+3	3		Loss of follow-up	0	0	0	0	1
PR56	4+4	4	45	Death by disease	0	0	0		0
PR57	3+4	2		Loss of follow-up					
PR58	5+4	5		No information	0	0	0	1	1
PR59	3+4	2	52	Loss of follow-up	0	0	0	0	1
PR60	4+3	3		Loss of follow-up	1	0	0	1	1
PR61	3+3	1		Loss of follow-up					
PR62	3+4	2		Loss of follow-up	1	0	0	0	0
PR63	3+3	1		Loss of follow-up	0	1	0	1	1
PR64	3+4	2		Loss of follow-up	1	0	0	1	0
PR65	3+4	2	10	Death by disease					
PR66	4+3	3	155	Loss of follow-up	0	1	0	1	0
PR67	4+3	3	43	Death without disease	1	1	0	1	1
PR68	4+3	3	158	Death by disease	1	1	0	0	1
PR69	4+4	4	28	Death by disease	1	1	1	1	1
PR70	3+3	1		Loss of follow-up	1	1	0	1	1
PR71	3+3	1	215	Live with disease	1	1	0	0	1
PR72	4+3	3		Live without disease - Loss of follow-up in 2008	0	1	0	0	1
PR73	3+3	1		Live without disease - Loss of follow-up in 2008	1	0	0		1
PR74	4+3	3	117	Death with disease (another cause)	1	1	0	1	1
PR75	4+5	5		No information	1	0	1	1	0
PR76	5+3	4	60	Death by disease	1	1	1	1	1
PR77	3+5	4	73	Death by disease	1	0	1	1	1
PR78	5+4	5		No information	1	1	1	1	0
PR79	4+3	5	213	Free from disease	1	0	1	1	1
PR80	5+5	5	38	Death by disease	1	0	0	1	1
PR81	5+4	5	70	Loss of follow-up	1	0	1	1	0
PR82	4+4	4	106	Death by disease	1	1	1	1	1
PR83	3+3	1		Loss of follow-up	1	0	1	1	1
PR84	3+3	1		No information	1	0	1	1	1
PR85	4+5	5		Live with disease	0	0	0	1	1
PR86	4+3	3		No information	1	0	1	1	0
PR87	4+3	3	173	Live with disease	1	0	1	0	1

PR88	4+5	5	29	Death by disease	1	1	1	1	1
PR89	4+3	3		Loss of follow-up	0	1	0	1	1
PR90	4+3	3	66	Death by disease	1	1	1	1	1
PR91	3+3	1		Live without disease - Loss of follow-up	1	0	0	1	1
PR92	3+4	2	178	Free from disease	0	0	0	1	1
PR93	3+3	1	45	Death by disease	1	1	0	1	0
PR94	3+3	1	103	Death without disease	1	0	0	1	1
PR95	4+3	3	24	Death by disease	1	1	1	1	1
PR96	3+4	2	148	Death by disease					
PR97					1	0	1	0	1
PR98	3+3	1	124	Live with disease	1	0	0	0	1
PR99	3+3	1		Loss of follow-up	1	0	0	0	1
PR100	3+4	2	173	Death without disease	1	0	0	0	1
PR101	3+4	2		Loss of follow-up	0	0	0	1	1
PR102	3+4	2	46	Death by disease	1	1	1	1	1
PR103	3+3	1		Loss of follow-up	1	1	1	1	1
PR104	3+3	1	175	Live with disease	1	1	0	1	1
PR105	3+4	2		Loss of follow-up	1	1	0	0	1
PR106	3+3	1		Loss of follow-up	1	0	0	0	0
PR107	3+3	1		Loss of follow-up	0	1	0	0	1
PR108	4+5	5		No information	0	1	1	1	1
PR109	3+3	1		No information	0	1	0	1	1
PR110	3+3	1	141	Live with disease	0	0	0	0	1
PR111	3+4	2	111	Live with disease	0	0	0	1	1
PR112	3+3	1	173	Free from disease	1	0	0	1	1
PR113	3+3	1	168	Free from disease	1	0	0	0	1
PR114	4+3	3	163	Free from disease	1	1	0	0	1
PR115	3+3	1		Loss of follow-up	1	1	0	0	1
PR116	3+4	2		Loss of follow-up	0	0	0		1
PR117	3+4	2	177	Free from disease	0	0	0		1
PR118	3+3	1	112	Free from disease	0	1	0	1	1
PR119	3+4	2	157	Death without disease	0	0	0	0	0
